# Supplementary material for: Red Arils of Taxus baccata L.—A New Source of Valuable Fatty Acids and Nutrients
Source: Molecules. 2021 Jan 30;26(3):723. doi: 10.3390/molecules26030723 (PMC7866497; doi:10.3390/molecules26030723)
Supplement: Supplementary file 1 [file molecules-26-00723-s001.pdf]

Red Arils of *Taxus Baccata* L.—A New Source of Valuable Fatty Acids and Nutrients

Małgorzata Tabaszewska [1], Jaroslawa Rutkowska [2,*], Łukasz Skoczylas [1], Jacek Słupski [1], Agata Antoniewska [2], Sylwester Smoleń [3], Marcin Łukasiewicz [4], Damian Baranowski [2], Iwona Duda [5] and Jörg Pietsch [6]

[1] Department of Plant Product Technology and Nutrition Hygiene, Faculty of Food Technology, University of Agriculture in Cracow, Balicka st. 122, 30-149, Cracow, Poland; malgorzata.tabaszewska@urk.edu.pl (M.T.); lukasz.skoczylas@urk.edu.pl (Ł.S.) jacek.slupski@urk.edu.pl (J.S.)

[2] Institute of Human Nutrition Sciences, Faculty of Human Nutrition, Warsaw University of Life Sciences (WULS-SGGW), Nowoursynowska st.159c, 02-776 Warsaw, Poland; agata_antoniewska@sggw.edu.pl (A.A.); damian_baranowski@sggw.edu.pl (D.B.)

[3] Department of Plant Biology and Biotechnology, Faculty of Biotechnology and Horticulture, University of Agriculture in Cracow, Al. 29 Listopada 54, 31-425 Cracow, Poland; sylwester.smolen@urk.edu.pl (S.S.)

[4] Department of Engineering and Machinery for Food Industry, Faculty of Food Technology, University of Agriculture in Cracow, Balicka st. 122, 30-149, Cracow, Poland; marcin.lukasiewicz@urk.edu.pl (M.Ł.)

[5] Department of Animal Product Technology, Faculty of Food Technology, University of Agriculture in Cracow, Balicka st. 122, 30-149, Cracow, Poland; iwona.duda@urk.edu.pl (I.D.)

[6] Institute of Legal Medicine, Medical Faculty Carl Gustav, Dresden Technical University, Fetscherstr. 74, D-01307 Dresden, Germany; joerg.pietsch@tu-dresden.de (J.P.)

* Correspondence: jaroslawa_rutkowska@sggw.edu.pl

Supplementary files:

Table S1. Comparison of fatty acid composition (g/100 g of FA) of red arils and seeds from Koszalin site (means ± SE).

Fatty acid	Red arils	Seeds
**SFAs**	**33.12 ± 0.31^{b}**	**4.87 ± 0.02^{a}**
C10:0	0.07 ± 0.00	ND
C12:0	0.30 ± 0.02	ND
C14:0	6.76 ± 0.04^{b}	0.05 ± 0.01^{a}
C16:0	22.37 ± 0.31^{b}	2.92 ± 0.01^{a}
C17:0	0.13 ± 0.00^{b}	0.04 ± 0.00^{a}
C18:0	3.37 ± 0.03^{b}	1.79 ± 0.00^{a}
C20:0	ND	0.07 ± 0.00
C22:0	0.11 ± 0.01	ND
C24:0	0.00 ± 0.00	ND
**MUFAs**	**13.04 ± 0.04^{a}**	**50.93 ± 0.03^{b}**
C14:1	0.15 ± 0.00	ND
C16:1 *n*-7	0.12 ± 0.00^{b}	0.03 ± 0.00^{a}
C16:1 *n*-9	0.26 ± 0.00	ND
C17:1	0.14 ± 0.01	ND
C18:1 *c9*	12.35 ± 0.06^{a}	50.15 ± 0.04^{b}
C18:1 *c11*	0.00 ± 0.00^{a}	0.52 ± 0.00^{b}
C20:1 *n*-9	0.04 ± 0.00^{a}	0.23 ± 0.00^{b}
***n-3*-PUFAs**	**24.18 ± 0.20^{b}**	**1.79 ± 0.02^{a}**
C18:3 *c9 c12 c15*	23.43 ± 0.23^{b}	1.79 ± 0.02^{a}
C20:3 *c11 c14 c17*	0.76 ± 0.02	ND
***n-6*-PUFAs**	**25.19 ± 0.16^{a}**	**26.84 ± 0.02^{b}**
C18:2 *c9 c12*	21.33 ± 0.11^{a}	25.69 ± 0.02^{b}
C18:3 *c6 c9 c12*	0.80 ± 0.02^{b}	0.43 ± 0.00^{a}
C20:2 *c11 c14*	0.16 ± 0.00^{a}	0.72 ± 0.00^{b}
C22:2 *c13 c16*	2.33 ± 0.04	ND
C20:4 *c5 c8 c11 c14*	0.57 ± 0.02	ND
**Δ5-UPIFAs**	**1.08 ± 0.01^{a}**	**15.20 ± 0.03^{b}**
C18:2 *c5 c9*	ND	11.08 ± 0.02
C18:3 *c5 c9 c12*	0.08 ± 0.00^{a}	1.53 ± 0.01^{b}
C20:3 *c5 c11 c14*	0.82 ± 0.02^{a}	2.34 ± 0.00^{b}
C20:4 *c5 c11 c14 c17*	0.17 ± 0.00^{a}	0.25 ± 0.00^{b}
**PUFAs**	**50.45 ± 0.16^{b}**	**43.83 ± 0.01^{a}**
NI	3.38 ± 0.45^{b}	0.37 ± 0.02^{a}

$^{a, b}$ – values baring the same superscripts in rows do not differ significantly ($p < 0.05$) from each other.

Abbreviations: Δ5-UPIFAs - Δ5-unsaturated polymethylene-interrupted FAs; C18:2 Δ *c9* - taxoleic acid; C18:3*Δ5c9c12* - pinolenic acid; C20:3Δ*5c11c14* - sciadonic acid; C20:4 *Δ5c11c14c*17 - juniperonic acid; ND not detected, NI – not identified.

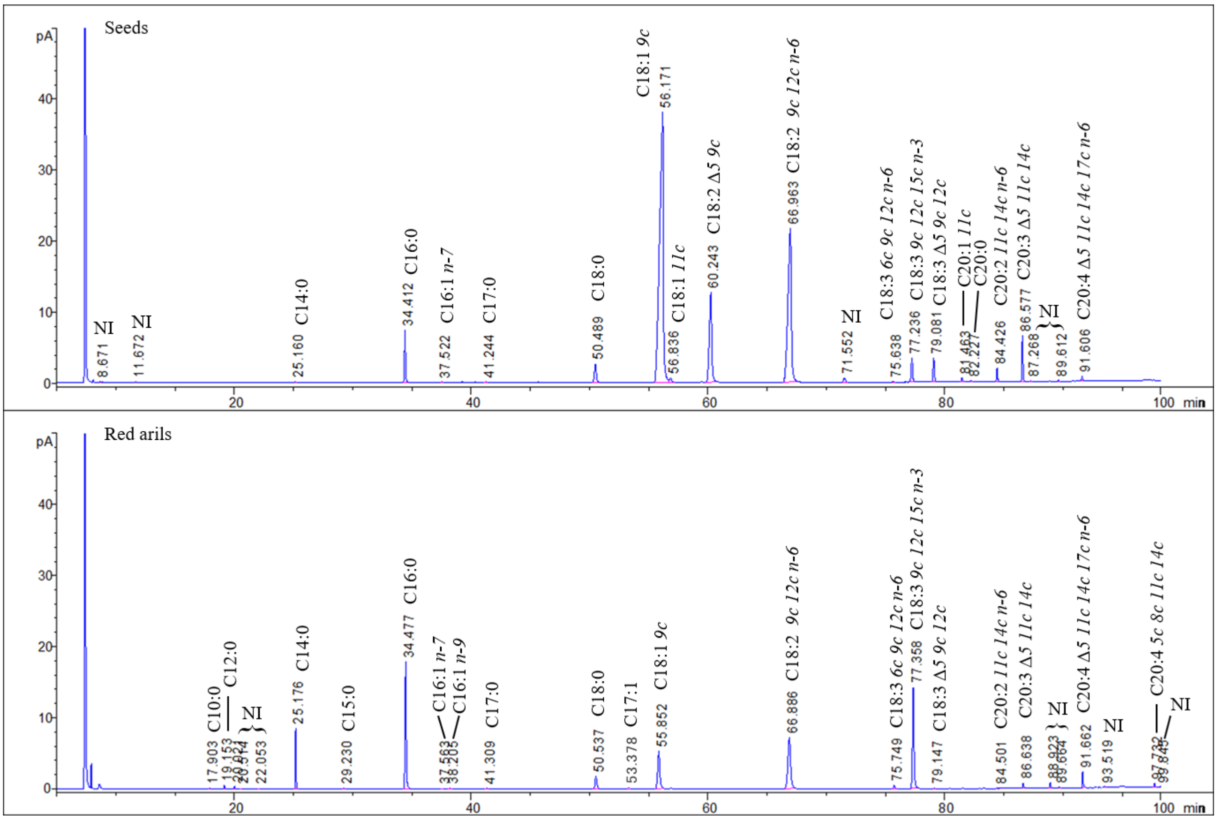

Figure S1. Separation of FAs composition of lipids extracted from seeds and red arils of *Taxus baccata* by gas chromatography.

Abbreviations: NI – not identified
